# A rare case of double ectopic thyroid in the superior mediastinum: a case report

**DOI:** 10.1093/jscr/rjad058

**Published:** 2023-02-22

**Authors:** Tejus V Nagireddy, Advait A Vaidya, Samir Gupta, Pankaj Kshirsagar, Tushar Kamble

**Affiliations:** Department of Surgical Oncology, Dr. D.Y.Patil Medical College, Hospital and Research Center, Dr D Y Patil Vidyapeeth, Pune 411018, Maharashtra, India; Department of Surgical Oncology, Dr. D.Y.Patil Medical College, Hospital and Research Center, Dr D Y Patil Vidyapeeth, Pune 411018, Maharashtra, India; Department of Surgical Oncology, Dr. D.Y.Patil Medical College, Hospital and Research Center, Dr D Y Patil Vidyapeeth, Pune 411018, Maharashtra, India; Department of Surgical Oncology, Dr. D.Y.Patil Medical College, Hospital and Research Center, Dr D Y Patil Vidyapeeth, Pune 411018, Maharashtra, India; Department of Surgical Oncology, Dr. D.Y.Patil Medical College, Hospital and Research Center, Dr D Y Patil Vidyapeeth, Pune 411018, Maharashtra, India

## Abstract

Primary ectopic mediastinal thyroid is rare, seen in <1% of patients with ectopic thyroid. A patient with two ectopic foci in mediastinum is even rare. Our patient presented with chronic cough and discomfort. CT scan revealed a huge 7 cm × 7 cm (right) and 5 cm × 5 cm (left) mass in mediastinum. IR-guided biopsy of the right side mass showed an ectopic thyroid tissue (ETT). Due to close proximity with major vessels, sternotomy is done and the two masses are excised. The masses were not connected in any way with each other as well as with the orthotopic thyroid in the neck. Pathology revealed colloid goiter. Surgical excision of a mediastinal mass is warranted. This helps in both the diagnosis and potentially be the primary treatment as well. Patients with ectopic thyroid disease are rare, and a presentation of two ETTs on both sides of mediastinum is very rare.

## INTRODUCTION

The most common mediastinal tumours are thymoma, neurogenic tumours and benign cysts, accounting for 60% [1]. Neurogenic tumours common in children; thymomas, thymic cysts and lymphomas common in adults [2]. Goiter often extend into retro-sternum, but primary ectopic thyroid in the mediastinum are very rare entities [3–5]. Aberrant thyroid can be found anywhere along the normal path of descent from foramen of cecum to anterior mediastinum. Undescended thyroid leads to lingual thyroid or the intemperate descent leads to mediastinal goiter. Surgical excision is occasionally necessary for these cases. Clinical literature pertaining to double ectopic mediastinal thyroid is very rare, as most of the patients are asymptomatic and present incidentally. Reporting a case of double ectopic mediastinal thyroid tissue resected through sternotomy approach. Though very rare, this case highlights the clinician and radiologist to consider ectopic as a differential when dealing with mediastinal mass, and the surgeon should consider the option of sternotomy as the size of the mass and close proximity to major vessels may lead to torrential bleeding when tried with neck incision alone.

## CASE PRESENTATION

**Figure 1:**
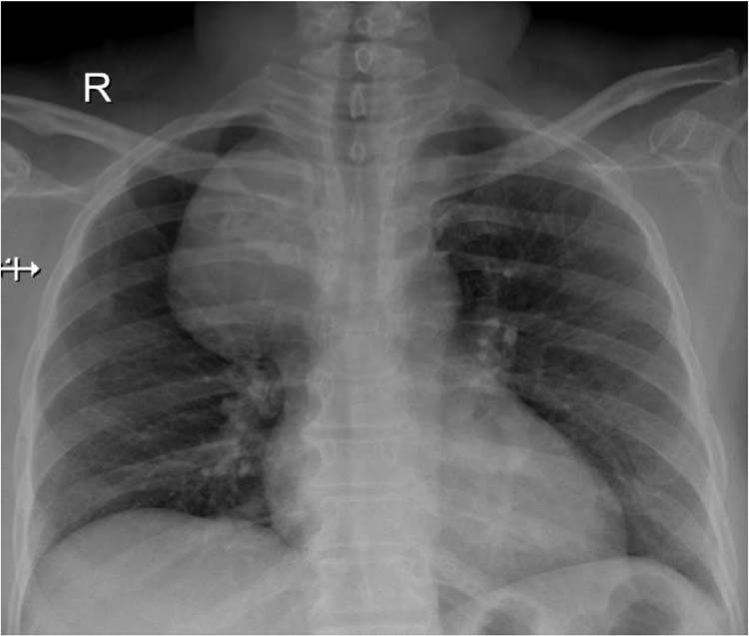
Chest X ray showing space occupying lesion in mediastinum.

**Figure 2:**
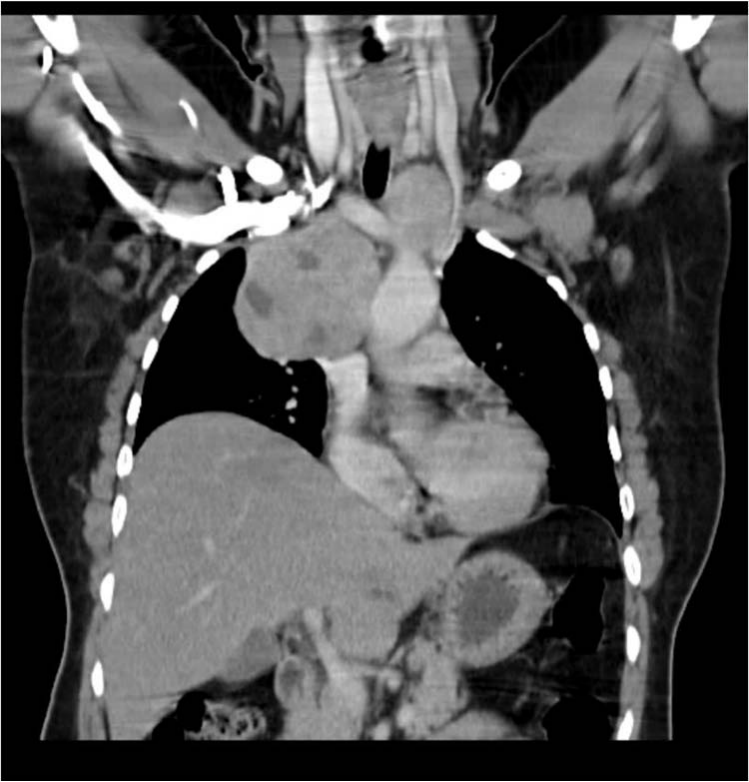
CECT scan showing the right side mass displacing superior nn(SVC).

**Figure 3:**
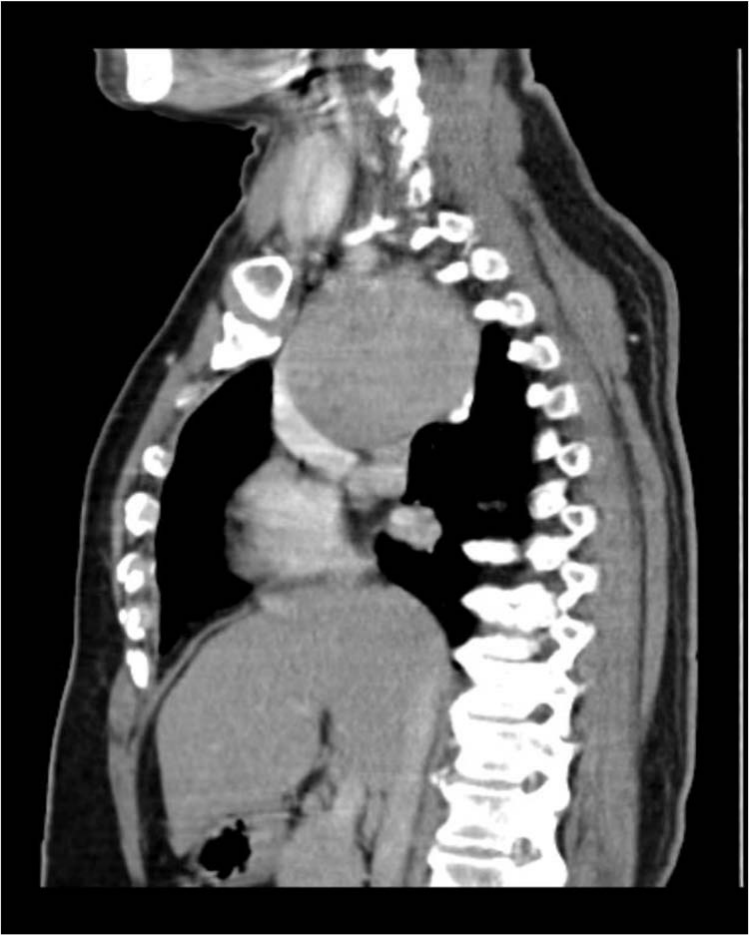
CECT scan showing mass.

**Figure 4:**
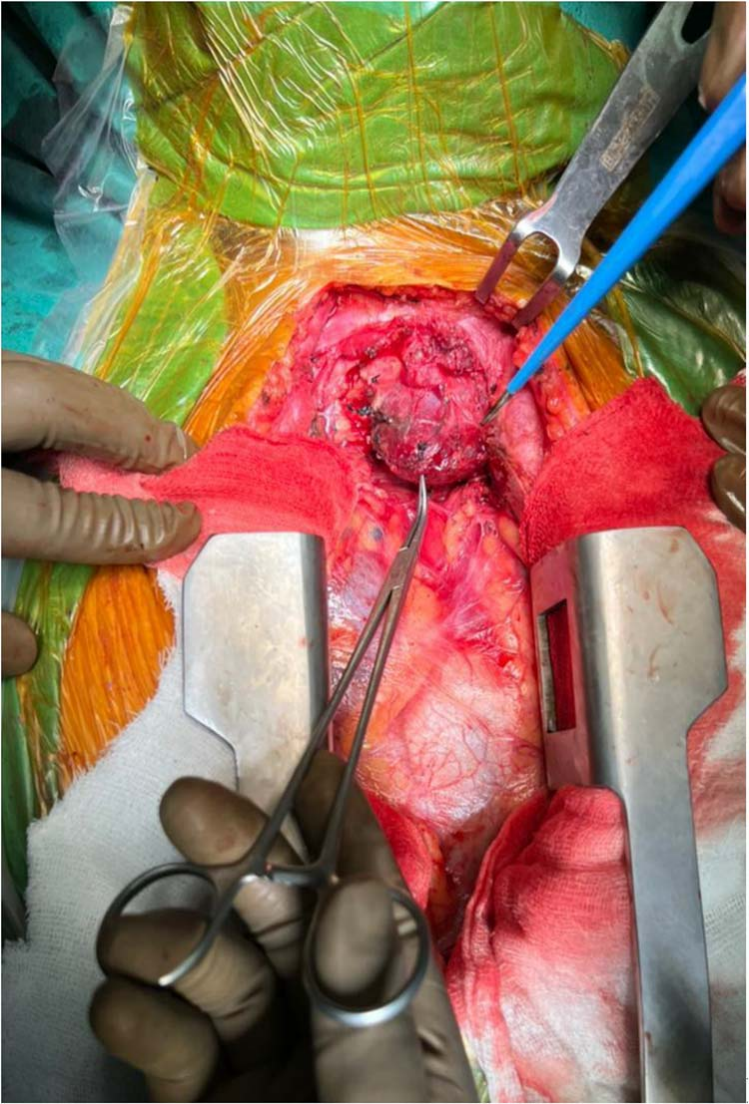
Encapsulated left side mass after sternotomy.

A 57-year-old female came with chest discomfort and cough since 1 month. The chest X-ray (Fig. 1) showed mass in the superior mediastinum. CECT thorax (Fig. 2) revealed the soft tissue density 7 cm × 7 cm (Fig. 3) in superior mediastinum abutting subclavian artery, azygous vein, anteriorly displacing superior venacava, anteromedially abutting arch of aorta, posteromedially compressing trachea and abutting right main bronchus. A 4.9 cm × 5 cm mass in the left side superior mediatinum was seen abutting internal jugular vein and brachiocephalic artery. USG showed the presence of normal thyroid in the neck. CT guided biopsy from the right mass showed ectopic thyroid tissue (ETT)-colloid goiter. Due to close proximity with major vessels sternotomy done. Mass in the left side 5 cm × 5 cm (Fig. 4) in the superior mediastinum was identified, on dissecting superiorly, which was free from the thyroid in the neck and abutting the IJV, brachiocephalic artery and left innominate vein that were separated with meticulous dissection.

**Figure 5:**
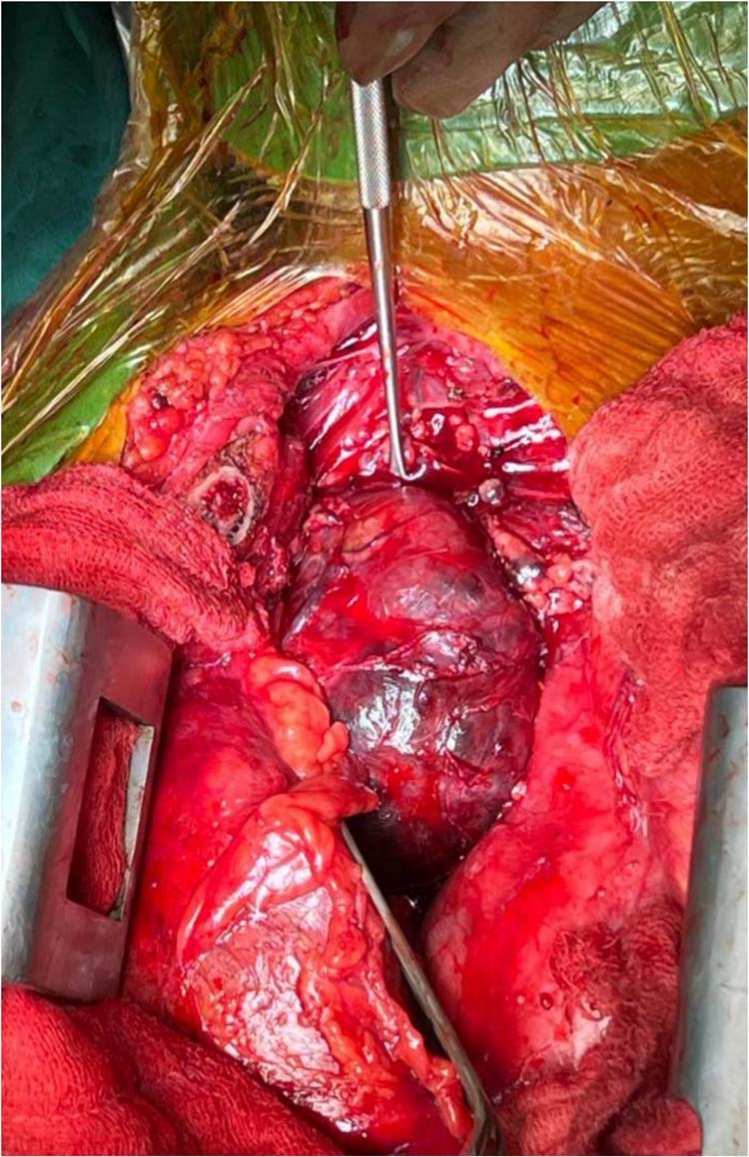
Right side mediastinal mass.

**Figure 6:**
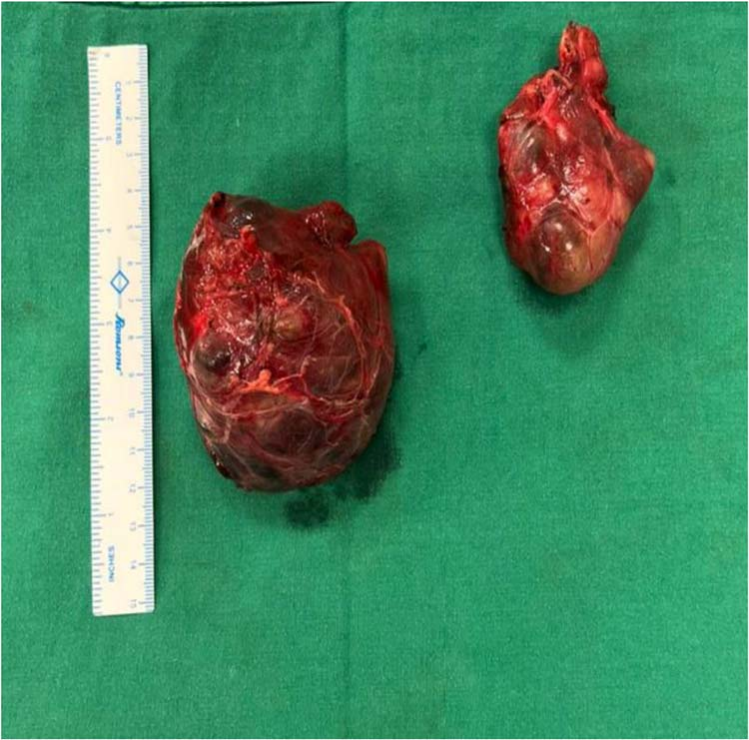
Mediastinal mass after excision.

**Figure 7:**
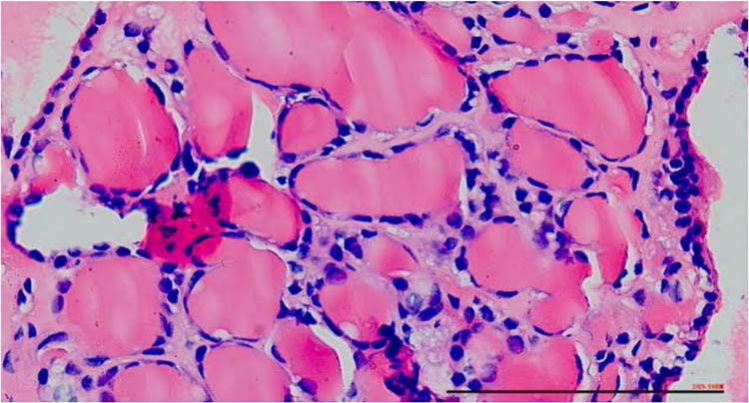
Excised mass with thyroid follicles.

**Figure 8:**
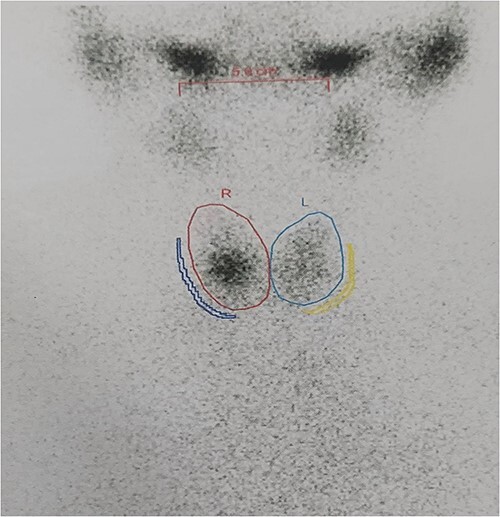
Thyroid scan showing ortho-topic thyroid with no uptake anywhere else.

The right mass (Fig. 5) was even bigger and needed an extension of incision into the neck (Fig. 6). About 7 cm × 7 cm noted to be displacing Superior venacava anteriorly that was dissected from it, separated from the first rib and subclavian artery meticulously taking care not to damage any major structure. Both masses were excised. These masses were not connected in any way with each other as well as with the orthotopic thyroid in the neck. Post-op was uneventful. Thyroid functions were normal. Thyroid scan (Fig. 8) showed no evidence of a thyroid tissue in the mediastinum and no other ectopic thyroid was present other than the orthotopic thyroid tissue in neck. The patient was discharged on POD 10 without any symptoms. The histopathological tissue diagnosis showed a colloid goiter (Fig. 7). All data kept in our database.

## DISCUSSION

Ectopic thyroids occur in one among 100 000–300 000 people [5]. In sum, 90% of ectopic thyroid glands are lingual thyroids, and the ETT present in the mediastinum, as in our case, accounts for less than 1% of all mediastinal tumours [6–8]. Santangelo *et al.* [9], in their retrospective analysis of all patients undergoing surgical treatment for thyroid disease, identified ETT in 0.9% patients and the anatomical site as follows: lateral cervical 21.4%, along thyroglossal duct 21.4%, mediastinal 17.9%, lingual 17.9%, sublingual 10.7% and submandibular 10.7%. As we mentioned, in our case- patient presented with double mediastinal localization of ETT with the simultaneous existence of a normal functioning TG which is a rare entity. Patients asymptomatic were usually found incidentally via imaging [14]. Compressive symptoms by the mass may occur [3, 15]. Cytologic results demonstrated an ETT, and, if not, our differential diagnosis would be to rule out thymoma and malignant lymphoma [2]. Thyroid scintigraphy may be a diagnostic test, but reported to be of value in patients without an orthotopic thyroid gland. CT remains the standard imaging to determine the size and extent of the mass and its relation to the surrounding structures [11, 12]. In the literature, a criterion has been defined for selecting patients for sternotomy based on CT features that include the volume of thyroid gland and its extension below the carina, the source of its blood supply and the risk of hemorrhage, and the presence of enlarged mediastinal lymph nodes as in the presence of malignancy [12]. Although 97% of mediastinal goiters can be delivered through a cervical approach, it is a wise decision to make with evidence of imaging to predict the need of sternotomy for the complete and safe resection [11]. Even with a combined cervicosternotomy approach, the prognosis of the patients in literature has been excellent with almost 0% mortality, resulting in immediate resolution of symptoms [10–12]. These separately capsulated masses, if attempted to pull through cervical incision, may possibly result in catastrophic bleeding with grave consequences. Management will depend on the final diagnosis, whether the lesion is benign or malignant. If a benign, low-risk lesion in an asymptomatic patient is found, there is an argument that could be made for regular imaging and watchful waiting. However, several authors who have treated patients with PMG state that surgical resection is the gold standard [14, 15]. Most authors state that primary mediastinal goiter is an indication for surgical resection. The first case of ETT was reported in 1869 by Hickman, and up to present, only 700 cases approximately of ectopic thyroid have been documented worldwide. Of interest, although ectopic thyroid in the mediastinum is rare, not many cases have been published. Table 1 shows a literature review by Metere et al [5] discussing mediastinal and other ectopic thyroid locations. Searching for double and triple ectopic thyroid, triple ectopic is found in some cases, as mentioned in Table 2. However, double ectopic in mediastinum alone as not been reported in the literature.

**Table 1:** Mediastinal ectopic thyroid recent publications.

Publishing year	Author	Country	Diagnosis	Title
2013	Roh *et al.* [13]	Korea	A case of mediastinal ectopic thyroid presenting with a paratracheal mass	Benign thyroid tissue
2014	Surng *et al.* [16]	Korea	A 7.3 cm × 5.3 cm × 3.5 cm heterotopic thyroid in the posterior mediastinum in a patient with situs inversus totalis	Posterior Mediastinal ectopic thyroid. benign thyroid tissue
2017	Hummel *et al.* [17]	USA	Ectopic thyroid tissue in the mediastinum characterised by histology and functional imaging with I-123 SPECT	Benign thyroid tissue
2017	Hu *et al.* [18]	China	Ectopic thyroid cancer diagnosed by endobronchial ultrasound guided transbronchial needle aspiration	Papillary thyroid carcinoma
2018	Raji *et al.* [18]	India	Ectopic thyroid: the great mimicker	Benign thyroid tissue
2018	Regal *et al.* [18]	Saudia Arabia	Mediastinal ectopic thyroid mass with normal thyroid function and location: case report	Benign thyroid tissue
2018	Metere *et al.* [18]	Italy	Diagnosis and management of a mediastinal ectopic thyroid laying on the right bronchus: case report and review of literature	Benign thyroid tissue.

**Table 2:** Double and triple ETTs

Publishing year	Author	Country	Title	Diagnosis
2021	Imai *et al.* [19]	Japan	A case of ectopic thyroid presenting as a mediastinal mass	Ectopic thyroid tissue with nodular goiter
2021	Muzurovic *et al.* [20]	Montenegro	Diagnosis and treatment of mediastinal ectopic thyroid tissue with normally located thyroid gland and primary hyperparathyroidism: a case report	Colloid goiter with cystic degeneration
2021	Kumar *et al.* [21]	India	Triple ectopic thyroid	Lingual, suprahyoid, infrahyoid
2011	Konde *et al.* [22]	India	Triple ectopic thyroid	Lingual, sublingual, infrahyoid
2013	Kuramoto *et al.* [23]	Japan	Triple ectopic thyroid	Near hyoid bone
2011	Nilegaonkar *et al.* [24]	India	Triple ectopic thyroid	Hyoid base of tongue and suprahyoid

## CONCLUSION

We consider that ectopic thyroid in the mediastinum, like in this case, should be differential. There are only few reported cases of surgical resection through a cervical incision, and these patients diagnosed with cervicomediastinal masses usually need multidisciplinary team approach in the setting of a tertiary care hospital. This case will highlight the importance of multidisciplinary approach and requirement of sternotomy as any attempt to deliver through cervical approach may lead to increased complications.
